# Address Given at the Buffalo Medical College, Introductory to the Course of Lectures for the Term Commencing Nov. 8, 1861

**Published:** 1861-12

**Authors:** J. R. Lothrop

**Affiliations:** Lecturer on Materia Medica


AND REPORTER.

VOL. I. DECEMBER, 1861. NO. 5.

### ORIGINAL COMMUNICATIONS.

ART. I.—*Address given at the Buffalo Medical College, Introductory to the Course of Lectures for the Term commencing Nov.* 8, 1861. *By* J. R. LOTHROP, *M. D. Lecturer on Materia Medica.*

GENTLEMEN OF THE MEDICAL CLASS:

The course of time brings us together to renew the consideration of subjects appropriate to this place. The swift years bear us on from one annual renewal to another, with a rapidity which seems accelerated as each succeeds the other, and which forcibly impresses upon us the brief period of opportunity in comparison with the vastness of the work to be done.—Those who in the years that have gone, have met here, came and went with the same purposes with which most of you have come and will go. A few who were here last year, have gone out, in this day of our country's need, to answer its requirements, with willing hearts, in a service which brings them among armed men, and it may be in the scenes of battle and of danger.

Our studies here which prosper best in the benign atmosphere of peace, yet fit us for services which war demands as well as peace, and while their general tendency is humanizing they are not inconsistent, as we have lately seen, with that heroism, which follows duty, though in the face of peril.—The distant mutterings of war and conflict, though they do not directly disturb our peaceful pursuits, agitate every loyal breast, and impress upon us a sense of duty to physical infirmity and suffering, which may lead to the very field of strife and danger. The influences which come from the great events now passing, strengthen the feelings which actuate every earnest professional man, to be true to the great purpose of his life, wherever it may lead, or whatever it may bring.

But, gentlemen, our winter's labors are before us, and whatever they may bring in the future, our present ambitions should relate to them. The field is broad, and though some have wrought longer and gained more than others, we are all students, and our objects and ends being the same, the distinction between older and younger is one of time and progress, rather than of aims. All are probably far behind the possibilities of unremitting study; for few make the most of themselves. But between him who has received his degree and him who has not, the distinction seems wide. The practical comes in to make the difference. It might be called the distinction between acquiring and applying, but this is not all, for though applying, the one is always acquiring, and the other while acquiring is in some sense applying. But there are to the practical physician considerations pertaining to his own personality, apart from his professional fitness, which the student does not experience, nor is he troubled with the uncertainties both as to diseases and remedies which the other encounters. He sees only the attractions of a science, which at every step of his progress presents new truths, and bears him on with a steady current of interest and novelty.

The mind is pleasantly excited with the daily acquisition of knowledge, which has almost the charm of discovery. As we climb the mountain at each step there opens upon the admiring sense a broader and more magnificent expanse—so the student as he rises in knowledge and his intellectual vision expands, experiences pleasures alike exciting and elevating. It is a delightful period of generous enthusiasm and emulation, which will ever be remembered in the more care-burdened period of life, as one productive of the best influences on the mind and character. It is well then that you should have it now, for your prime object, to become acquainted with the scientific rather than the practical part of medicine, and you will find that to him who is in earnest, in his work, science is not chary of its grand and fascinating truths.

In our admiration of the vast and imperishable structure which modern intellect, research and industry have reared, we are apt, in our exultation, to give ourselves over to the feeling that we are close on to perfection, and to rest upon what is already known. Such a feeling is too strikingly at variance with the whole spirit of medicine, which is so nobly progressive, and can bring nothing but injury to it. Such complacent partiality is not less destructive to progress than indolence or obstinacy, for it is as bad to feel that there is nothing to learn, as to waste or reject the opportunity to do so, as either tends to stagnation of mind. Besides being harmful and unprogressive in its tendency, this notion of perfection is wholly unfounded, our knowledge in some of the departments of medical science being as yet very incomplete.

But before we take into consideration the present state of the science, it will not be without interest to speak of its progress from the earliest periods. As we go back we do not find much that is definite before the time of Hippocrates, 400 years, B. C. Whether it was the excellence or the absence of the medical art which gave to the antideluvians their length of days, is more than can be now stated. Even after the flood, in those nations which kept the feeble fire of knowledge alive, we find but slender traces of any system of medicine. The medical art consisted mainly in incantations and religious ceremonies. There was a belief in charms, and amulets were worn not as in modern times for ornament, but from a belief in their health-giving powers. The stars were thought to be potent over the diseases as well as the destinies of men, and they invoked the aid of astrologers to read their portents, if not to avert their power. Some rude art there probably was, some vague idea of disease and some limited use of remedies, but as disease was believed to be due to supernatural causes, as among the Greeks to the anger of the Gods, so the means of averting and curing it were of a nature suited to the belief of its origin, such as charms, rites and superstitious observances. We could not look for progress in the midst of so much superstition. The efforts of Esculapius were undoubtedly altogether human, and he probably brought some remedial means, other than mere mystical rites to bear on disease; and all the fables of his divine origin sprang from the prevalent belief, that as disease was of divine origin, divine agency could alone cure or avert.

The Jews added but little to medical science, and even the very wisdom of Solomon was not above the use of charms. Hippocrates first gave form to medicine and furnished it with a set of rules—though his works, amidst much that is practical, contain a great amount of worthless and erroneous matter—"exceptional truths, vulgar observations, even errors and contradictions." But it is a proof of his great genius, that he obtained a complete authority over the medical mind of succeeding ages, and he has given some lessons in observation of disease which have never been surpassed.—Great as his sagacity and genius must have been, he probably was not independent of the labors of some who had preceded him, for the great minds of any age are not wholly original, and create the knowledge which they leave; there are always some humbler labors, or some antecedent intellectual tendencies which give aid and direction. But still the beginning of our science must date from him. The medical mind took its direction from him, and some centuries later, 200 A. D. Galen taught his doctrines and extended his authority, in fact, assumed his place and exercised an authority almost absolute over medicine for 1200 years. During the period between these two great men, the science of anatomy was considerably advanced, though most works of the time were lost by the burning of the Alexandrian Library. Galen taught and wrote on anatomy, physiology and hygiene. He had some idea, though not a true one, of the motion of the blood, for he believed the veins carried the blood from the heart, and that the ventricles communicated directly. He was altogether a man of great genius and profound learning. The best evidence of this is, that men of undoubted intelligence taught his doctrines, unfounded as they were, with entire conviction of their truth, and not till anatomy began to be more thoroughly studied, and men began to see with their own eyes, did this unquestioning acquiescence cease, and his influence wane. Those who followed him for centuries, were mere imitators, and added nothing; in fact, it was fortunate if medicine maintained its state, in the midst of the darkness and mental stagnation which succeeded. Bacon in his time, called medicine, rather circular than progressive, for he found great repetition and but little new matter in the writers of physic.

We can, in view of the freedom of thought and the spirit of inquiry which characterize our times, with difficulty conceive of the courage which was necessary to encounter the fixed beliefs of the time, so that even Harvey hesitated to publish his discovery, fearing the assault and ridicule which it actually met. It is a wonderful instance of mental servility, and of the pernicious influence of an error once taken for granted, that men saw the opening between the ventricles of the heart which Galen asserted to exist. Till within 300 years there was very little medical inquiry, and for a century previously, false science prevailed, extravagant and wild theories arose, and sects founded upon some single notion of the nature of disease or the operation of remedies absurd and erroneous, existed. They were many of them feeble and unprofitable protests against the dominant doctrines.

Such were the wild dreams of alchymy—searching for the philosopher's stone and the elixir of life, the former of which afforded but little satisfaction to the seekers, and the latter proved of but little value when claimed to have been found, for Paracelsus, who professed to have discovered it, is said to have died with a bottle of it in his pocket. One of the alchemists has left the following record of his experience in the search,—" having lost the time and money which you have devoted to it, you will find yourself old, ragged, hated, famished, always smelling of sulphur, soiled with sweat and charcoal,—paralytic with frequent manipulations of quicksilver—gaining nothing but a running nose—in a word, so unhappy that you will be willing to sell your body, and even your soul." But this period of imitation, ignorance, and intolerance began gradually to disappear, and a more liberal and enterprising spirit was brought to the study of medical science. In the fourteenth century anatomy began to be studied with more accuracy and a true knowledge of the structure of the body to be gained, and in this and the following century, many discoveries were made. During this time medicine has among its great names, those of Varolius, Sylvius, Fallopius, Eustachius, Fabricius and Vesalius. The last named, published a great work on anatomy in 1543, in which he was the first to dare the assertion that no opening existed between the ventricles, and was consequently greatly abused. The names of the others are familiar to all students, associated with parts of the body which they first described. In the next century, Harvey announced his great discovery, and medical science may be said to have had a permanent existence. Since then it has gone on with an ever increasing accumulation of discoveries and facts, to its present advanced and perfected state—not complete but enriched with a vast amount of knowledge in its various departments, and with a spirit so liberal, inquisitive, and progressive, that there is reason for an assurance that it will be freed from its uncertainties and be attended with more definite and useful results.

Time will permit to speak only in these general terms of the progress of medicine during the last two centuries. An enumeration of the many discoveries in every department, and a mention of the illustrious names which adorn it, would require more space than is allotted to this brief address.—It is a brilliant period which it is pleasant to contemplate. From what has been said it is apparent that in the beginning medicine was a mere art, and did not and could not become a science till individual facts enough had been collected and arranged to warrant some rules to express what was common to them all. But the art was mere empiricism, and not like the medical art which now springs from science, and is but the practice of its rules.—It is apparent too, that like all sciences, it has been influenced by the particular intellectual direction of the age. The early ages of the world were full of faith in the fabulous, and medicine was full of mysticism. The speculative Greek imparted to it the learning of the period which begun with Hippocrates and ended with Galen, and made it largely theoretical.—The long night of the dark ages, rather took from, than added to, what the ancient learning had given it, and when the general mind awoke from the night of the middle ages, it felt the stimulus of awakened intellect. Then printing was invented, a new world was discovered, Luther preached, Copernicus announced the solar system, with the sun for its centre, and later, Bacon wrote. By the spirit of religious freedom and the growth of commerce, the intolerance which had weighed heavily on all philosophy and science was shaken off, and men observed and thought with some independence of antecedent authorities. The present is the period of positive science, and most of the departments of medical science are entered upon it. But it is yet a great way from perfection. It cannot be called perfect till the structure and function of every tissue and organ of the human body shall have been ascertained, and the agents which prevent or remedy all deviations from healthy structure, or diseased functions, shall have been discovered. The former involves the perfection of anatomy and physiology, and the latter of therapeutics. But physiology has not yet shown the function of every organ. It has not yet, perhaps, wholly shaken of the tendency to explain the phenomena of vitality, by the laws which govern merely physical actions; and by forces which are seen to operate on the world of matter around us. It is perhaps too much inclined to bring vital operations, within the sphere of chemical action; but its progress has been truly wonderful, and from the labors of the earnest and truth-seeking men who are now giving their minds to it, we may expect greater results. There can be no doubt that from the direction which enquiry is now taking, and the methods of investigating phenomena, new discoveries will be made which will overthrow much that has been hitherto considered certainly known. Closely allied to it in its progress, and governed by it, is pathology, which treats of diseased functions, and as our knowledge of organization and repair which are morbid in character, must depend upon that of health—therefore it must keep pace with physiology, though it is not deducible from it, for knowing a healthy function will not lead us to infer correctly what will be its morbid action.

But therapeutics or the treatment of diseases is far from that perfect state, when an agent is known for every diseased action. Its progress is wholly the result of experiment, and as we arrive at particular facts only by repeated observations, we very often find a want of accuracy, or of number in the particular observation which establish them, and many statements pass for facts which have no other foundation than fallacious experience.—We have something to learn of the natural history of diseases, and we are certainly very far from having a perfect understanding of the application and operation of remedies. This brief statement is enough to show the present imperfection of our science, and to show moreover, that the deficiency is in one particular, one that all can assist in removing, for in the establishment of any fact, the larger the number of cases the nearer we approach to certainty. Carefully observed and well arranged cases are important in establishing positive laws of disease, and are within the powers of the great majority of medical men. We must not therefore, accept the idea of perfection and dismiss from our minds the necessity of labor for its further advancement.

But this statement of the progress and present state of medical science, though it is calculated by showing the deficiencies of our present knowledge, to preserve us from that boastful spirit to which there is some tendency—affords us the truest satisfaction, in the reflection that all in our knowledge which has proved of permanent value, which has stood the test of time, and will endure, has been the work of the students of regular medicine.

The various theories which have from time to time claimed attention, have mostly been forgotten, many of them were extravagant—some of them, for the time, widely influenced even intelligent minds—but they have passed away. They depended upon interest or delusion in the originator, and were not the offspring of that love of knowledge for its own sake, which marks the scholar. One, as we believe, erroneous system of comparatively modern origin, still maintains itself extensively in this country, though in the old world, the land of its birth, its influence is on the wane.

At this point the question naturally arises, as our knowledge extends, and undoubted progress is made, as we become more thoroughly acquainted with the laws of healthy and diseased action, is there also corresponding progress in our means for the prevention and cure of disease—the ultimate and final purpose of all our labors. To this it can with truth be replied, that our means of preventing disease and alleviating suffering have very greatly improved, and it is due partly, no doubt, to improved discrimination, but largely also to improved methods. There is positive evidence that diseases now are cured by both medical and surgical means, which in times past were either very rudely, or not at all cared for. But with all our improvement in treatment, the advance has not been corresponding, for there is no doubt medical science is more advanced than medical art, and that diagnosis is superior to treatment. It is easier to perceive the morbid condition of an organ, or function, than to find means to prevent or cure. Our difficulties are much greater in establishing definite results of treatment, than laws of disease. But in this there is much to encourage, for though the merest empiricism may be often quite successful in dealing with particular diseases, accuracy of diagnosis is essential to rational treatment, and more likely to lead to profitable results.

In the foregoing remarks I have chosen to consider medicine from the scientific rather than the practical side. I hope I have succeeded in showing you the importance of thorough acquaintance with the science. You will not then be inclined to accept the assertion of those who declare, that knowledge of anatomy, physiology, and pathology, is not necessary for the successful practice of medicine. You will, I trust, perceive that an acquaintance with these branches lies at the foundation of your education, for till you are familiar with the structure and functions of the body and their deviations, there can be no sound reasoning or practice. At the beginning of the study of a science which embraces a great variety and amount of knowledge, it requires some judgment to know what not to learn, as well as what one can most usefully acquire—it can be answered generally, study the science before the art, diagnosis before treatment, healthy function and structure before that which is morbid, the general properties of medicines before their special uses.

I would willingly say more in this connection, but I have only time to say a few words on the importance of observation. Your opportunities here include some clinical study. Whatever real benefit they afford will come from the use of your faculties of observation, viz: your senses and your understanding. The use of the senses—the quick eye, and the educated touch are not the whole of it, it needs the mind to guide them. The thinking and the perceptive faculties are both requisite to a good observer. Touch, auscultation and percussion, the microscope, chemical examinations, autopsies, give us isolated facts, but the mind must collect and arrange them before they give us positive laws. But though the senses are not sufficient for true observation, it is quite necessary that they should be rightly used, so that what is seen may be really seen, and what is felt really felt—for it is not every man who sees or hears rightly. The mind, so to speak, is in the eye, or the ear, or even at the fingers' ends, and these senses instead of informing the mind truly, give it the information it wishes to receive. It is not in this last sense that we are to understand the wise advice of Dr. Latham to his pupils, in the wards of St. Bartholomew's—"it is with your own eyes, and your own ears, and your own minds, and (I may add) by your own hearts, that you must observe, and learn, and profit."

It will be well then to improve every opportunity which offers to observe the course of disease, uninfluenced by remedies when you can, but at any rate arm yourselves as well as you can with those methods of observation, which in any one case cover the whole ground, and leave nothing for any explorer who may come after. It is a want of such thoroughness and discrimination, which is fruitful of failure to many medical men, and fills our science with a host of false facts. One of the causes of the uncertainties of the numerical method of which we hear so much at present, is the utterly untrustworthy observations which provide the separate facts, for to obtain any reliable results we must regard their *quality* as well as their number.

In conclusion, let me say a few words of the spirit and method which should animate your studies, not only while you follow them here, but throughout your whole professional life, for habits early formed, will have a great influence over your later years. Let me urge you to be thoroughly in earnest, for earnest work is the best work in the world, and whatever direction it takes will make itself felt. The heart should be in the work in order to enlist the whole man. We are never laggards in the tasks we love. Our science has never lacked earnest men, who have exhibited in its pursuit the most sublime heroism. Devotedness is a mighty agency in the world's affairs.

Akin to this is thoroughness. A thing once well learned is worth many partly learned. It is better to go to the bottom of one branch of knowledge, than to skim over the surface of all the branches. In medicine superficial attainments are productive of mischief. If you are not willing to go deeply into a subject, you must ever fall below, even unhonored mediocrity. Thoroughness is the offspring of persistence of purpose which is one element of greatness. Moreover, the doing of one thing well, leads to the habit of doing many things well, the fruits of which in a life-time can hardly be stated. It will not allow you to take knowledge at second hand, but from the original sources. It is the companion of originality, for there can be no certain originality when the works of others have not been gone over. I am certain that as a class, medical men are as thorough as any professional men, and cultivate as largely, all the powers of the understanding. Dr. Johnson praised them for their general culture. We should strive to merit his commendation when he writes—"the physician is more apt to cultivate all the powers of his understanding, and all the departments of nature together; and has, therefore, been more distinguished for an enlightened and comprehensive view of the various subjects for reason, than any other class of mankind."

The scientific studies make men jealous for truth, and hence they are termed skeptical. Natural science liberalizes the mind ; but inasmuch as its truths will bear examination, it begets the habit of questioning all propositions which present themselves for acceptance, but questioning is not always unbelief.

But, gentlemen, to pursue a science in earnestness and thoroughness is no light thing—to be entered upon with a feeble purpose, which soon gives o'er. It requires days and nights of persevering study—systematic industry, careful appropriation of time, and above all, a love of it. Nature remains constant, the laws of life and the mysteries of the human frame were the same 2000 years ago. The capacity for knowing was the same, 'twas the finding out, which was so long wanting. Indeed the capacity for knowing is greater now than what is already found out, therefore, because we can know more, more will be known. We are all anxious to help on the progress of our science, which we can do by endeavoring to find out something new; but let us beware of standing in the way and hindering rather than assisting. By accepting as truth what is not true and adhering to it, we retard its progress. There is no science or department of knowledge which has not felt this retarding influence. We have but to remember how many centuries, the sun and all the company of stars were thought to make a daily revolution around this earth, till Copernicus found out their true motion. Let us question closely the beliefs of to-day, lest we cherish opinions which shall prove obstacles to the progress of science.

---

The Medical Director of the Army desires that exsection of the shoulder and elbow-joints shall be resorted to in preference to amputation, in all cases offering a reasonable hope of success, and that Pirogoff's operation at the ankle should be preferred to Chopart's, or to amputate above the ankle, in cases that might admit of a choice.

